# Pulse Wave Velocity Is Associated with Increased Plasma oxLDL in Ageing but Not with FGF21 and Habitual Exercise

**DOI:** 10.3390/antiox9030221

**Published:** 2020-03-07

**Authors:** Shuen Yee Lee, Stephen F. Burns, Kenneth K.C. Ng, David J. Stensel, Liang Zhong, Frankie H.Y. Tan, Kar Ling Chia, Kai Deng Fam, Margaret M.C. Yap, Kwee Poo Yeo, Eric P.H. Yap, Chin Leong Lim

**Affiliations:** 1 Lee Kong Chian School of Medicine, Nanyang Technological University, Singapore 308232, Singapore; slee066@e.ntu.edu.sg (S.Y.L.); karling.chia@ntu.edu.sg (K.L.C.); kdfam@ntu.edu.sg (K.D.F.); margaret.yap@ntu.edu.sg (M.M.C.Y.); ericyap@ntu.edu.sg (E.P.H.Y.); 2 Physical Education and Sports Science Academic Group, National Institute of Education, Nanyang Technological University, Singapore 637616, Singapore; stephen.burns@nie.edu.sg; 3 Novena Heart Centre, Singapore 329563, Singapore; kennethng@novenaheartcentre.com.sg; 4 National Centre for Sport and Exercise Medicine, School of Sport, Exercise and Health Sciences, Loughborough University, Leicestershire LE11 3TU, UK; D.J.Stensel@lboro.ac.uk; 5 National Heart Research Institute Singapore, National Heart Centre Singapore, Singapore 169609, Singapore; zhong.liang@nhcs.com.sg; 6 Duke-NUS Medical School, National University of Singapore, Singapore 169857, Singapore; 7 Sport Science & Medicine Centre, Singapore Sport Institute, Sport Singapore, Singapore 397630, Singapore; frankie_tan@sport.gov.sg; 8 Department of Physiology, Yong Loo Lin School of Medicine, National University of Singapore, Singapore 117593, Singapore; 9 School of Physical and Mathematical Sciences, Nanyang Technological University, Singapore 637371, Singapore; kweepoo@ntu.edu.sg

**Keywords:** exercise, ageing, oxLDL, arterial stiffness

## Abstract

Fibroblast growth factor 21 (FGF21) and adiponectin increase the expression of genes involved in antioxidant pathways, but their roles in mediating oxidative stress and arterial stiffness with ageing and habitual exercise remain unknown. We explored the role of the FGF21–adiponectin axis in mediating oxidative stress and arterial stiffness with ageing and habitual exercise. Eighty age- and sex-matched healthy individuals were assigned to younger sedentary or active (18–36 years old, *n* = 20 each) and older sedentary or active (45–80 years old, *n* = 20 each) groups. Arterial stiffness was measured indirectly using pulse wave velocity (PWV). Fasted plasma concentrations of FGF21, adiponectin and oxidized low-density lipoprotein (oxLDL) were measured. PWV was 0.2-fold higher and oxLDL concentration was 25.6% higher (both *p* < 0.001) in older than younger adults, despite no difference in FGF21 concentration (*p =* 0.097) between age groups. PWV (*p* = 0.09) and oxLDL concentration (*p =* 0.275) did not differ between activity groups but FGF21 concentration was 9% lower in active than sedentary individuals (*p* = 0.011). Adiponectin concentration did not differ by age (*p* = 0.642) or exercise habits (*p* = 0.821). In conclusion, age, but not habitual exercise, was associated with higher oxidative stress and arterial stiffness. FGF21 and adiponectin did not differ between younger and older adults, meaning that it is unlikely that they mediate oxidative stress and arterial stiffness in healthy adults.

## 1. Introduction

Arterial stiffness is an independent risk factor for cardiovascular mortality and is involved in various metabolic diseases [1]. Arterial stiffness, evaluated using arterial tonometry pulse wave velocity (PWV), increases exponentially with normative ageing, even within a healthy population [2]. The ageing process independently contributes to the development of arterial stiffness, leading to functional changes in major arteries [3]. Age-associated arterial stiffness is mainly due to an increase in oxidative stress [3]. In clinically healthy middle-aged men, a higher circulating concentration of oxidized low-density lipoprotein (oxLDL), a marker for oxidative stress, was associated with a four-fold higher risk of future coronary heart disease [4]. OxLDL concentration was also positively related to carotid and femoral artery intima-media thickness and plaque occurrence, which promotes arterial stiffness [4].

While ageing is associated with increased arterial stiffness, exercise training is effective for ameliorating age-associated arterial stiffness [5]. Compared to their sedentary counterparts, habitually active individuals have 20–35% lower arterial stiffness [6]. Exercise improves arterial stiffness mainly through a reduction in oxidative stress. For example, two to three sessions (>30 min each session) of weekly exercise improved total arterial compliance [7] and arterial compliance improved by 28% to the level observed in endurance-trained individuals after infusion of antioxidants in sedentary individuals [8].

Fibroblast growth factor 21 (FGF21) is a novel hormone that is predictive of cardio-metabolic disorders [9]. FGF21 protects against cardiac hypertrophy, cardiac dilation and cardiac dysfunction in animal models [10]. However, people with cardiovascular diseases have increased resting concentrations of circulating FGF21 [11], suggesting either a compensatory cardioprotective effect of FGF21 or FGF21 resistance in cardiovascular disease states. FGF21 concentration is positively associated with carotid intima-media thickness and PWV in patients on haemodialysis [12] and patients with type 2 diabetes [13]. These results imply a potential link between FGF21 and arterial stiffness, possibly through the moderation of oxidative stress. We speculated that FGF21 could improve changes in arterial stiffness associated with ageing and exercise by reducing oxidative stress. For example, both in vivo and in vitro administration of FGF21 in animals increased the expression of genes involved in antioxidative pathways, including mitochondrial uncoupling proteins (Ucp2 and Ucp3), superoxide dismutase-2, and decreased oxidative stress mediated by reactive oxygen species production [14,15]. Protein and mRNA expression of FGF21 also increased in cultured cardiac endothelial cells and hepatocytes treated with oxLDL [16,17].

Chronic FGF21 administration in both humans and mice increased plasma adiponectin concentration in a dose-dependent manner [18,19]. Further following myocardial infarction, adiponectin-null mice receiving FGF21 treatment had a blunted FGF21 response, which resulted in decreased capillary density, increased myocyte apoptosis and increased proinflammatory cytokine expression [20]. FGF21-deficient mice administered with adiponectin also exhibited alleviated atherosclerotic plaque formation [21]. These results suggest that adiponectin may be a downstream effector of the FGF21 pathway and that the FGF21–adiponectin axis may mediate the cardioprotective effects through the upregulation of genes involved in antioxidant pathways to improve arterial stiffness. Unlike FGF21, the links between adiponectin and arterial stiffness have been studied [22]. Adiponectin concentrations in hypertensive patients are negatively associated with PWV and plasma adiponectin concentrations independently predict arterial stiffness progression [23]. Adiponectin could influence arterial stiffness through the associated increase in antioxidant activity, which increases vasodilation and reduces systemic oxidative stress [24]. FGF21 and adiponectin concentrations increases with ageing, even in healthy individuals [25,26]. However, whether the FGF21–adiponectin-related increase in the expression of genes involved in antioxidative pathways is associated with the regulation of arterial stiffness, and its interaction with habitual exercise and ageing, is not known. Therefore, the aim of this study was to explore the associations between the FGF21–adiponectin axis with oxidative stress and arterial stiffness, under the influence of ageing and habitual exercise. We hypothesised that age-related increase in FGF21 is associated with an increase in oxidative stress and arterial stiffness. We also hypothesised that a habitual exercise-related decrease in FGF21 is associated with a decrease in oxidative stress and arterial stiffness.

## 2. Materials and Methods

### 2.1. Participants

Eighty participants from the Exercise for Life across Asia (ELIXA) cohort were recruited and assigned to four equal groups (*n* = 20 each) based on age and exercise history: Younger (18–36 years old) Active (YA) and Sedentary (YS), and Older (45–80 years old) Active (OA) and Sedentary (OS). The active and sedentary subgroups within each age group were matched for sex and age, in five-year intervals. Exercise profiles of the participants were based on self-declared five-year history of exercise participation (type, duration, frequency and intensity). The YA and OA groups participated in ≥3 sessions/week of moderate to vigorous intensity aerobic exercises for ≥45-min/session. These exercise participation criteria were within the recommended exercise guidelines of 75–150 min/week for moderate-to-vigorous intensity exercise [27]. The YS and OS groups engaged in <2 sessions/week of light intensity aerobic exercises, lasting <30 min/session. Exercise intensity was classified using descriptors that were based on the talk test [28] and breathing or heart rate responses, according to the global physical activity questionnaire [29]. The participants selected their individual exercise intensity based on these descriptors and the research team was available to provide guidance if needed. Light intensity exercises were described as activities causing a slight increase in breathing or heart rate, with participants being able to talk comfortably while doing the activity. Moderate to vigorous intensity exercises were described as activities requiring moderate to hard physical effort, causing larger increases in breathing or heart rate, with participants being unable to talk comfortably while doing the activity. The exercise profile was calculated by multiplying the sessions per week by the time spent per session for each exercise, and the total sum was reported as weekly exercise duration. The participants were non-smokers and non-habitual alcohol drinkers and female participants were not on oral contraceptives or hormone replacement therapy. All the participants were assessed and documented to be “healthy” (free from disease, medication and treatment) through medical screening by an appointed clinical service provider (Raffles Medical Group, Singapore). None of the participants recruited were obese or in the high-risk category of body mass index (BMI) for Asians (i.e., BMI was ≤27.5 kg/m^2^ for all participants) [30]. The medical screening included family history of diseases, assessment of blood pressure, height, weight, vision, urinalysis, and physical examination. The participants were recruited through posters, social media, and community outreach events. The participants were briefed on the nature and risks involved in the study and their right to withdraw their participation. All subjects gave their written informed consent for inclusion before they participated in the study. The study was conducted in accordance with the Declaration of Helsinki, and the protocol was approved by the institutional review board of Nanyang Technological University (IRB-2015-05-029).

### 2.2. Anthropometry and Blood Sample Collection

The participants abstained from alcohol, caffeine and dietary supplementation, kept to their regular diet and sleep routines for 24 h and refrained from physical exercise for 48 h before their visits to the laboratory. On the day of the trial, participants arrived between 0830 and 0930 h after an overnight fast and declared that they were well to proceed with the trial procedures and had their blood pressure (BP) measured (UM-102, A&D Medical, Tokyo, Japan). Pulse pressure was calculated as the difference between systolic and diastolic BP. Nude body weight was measured using an electronic scale (Seca 803, SECA, Hamburg, Germany) and height was measured using a stadiometer (Seca 217, SECA). BMI was calculated as body mass (kg) divided by height (m) squared. Waist circumference (WC) was recorded using a Gulick tape (Seca 201, SECA, Hamburg, Germany) which was placed snugly at the waistline, midway between the lowest ribs and iliac crest in a standing position. WC was measured thrice, recorded to the nearest 0.1 cm in triplicate and averaged from the measurements.

A baseline venous blood sample was collected from the forearm into serum and dipotassium ethylenediaminetetraacetic acid (K2 EDTA) vacutainer tubes (4 mL) and placed on ice (4 °C) immediately. Fasted serum (4 mL) were analysed for triglycerides (TG), total cholesterol (TC), high-density lipoprotein cholesterol (HDL-C) and low-density lipoprotein cholesterol (LDL-C) levels (Siemens ADVIA 1800 and ADVIA Chemistry XPT, Berlin, Germany) by a licensed pathology laboratory (Quest Laboratories, Singapore, Singapore). Fasted glucose concentration was measured immediately after the blood sampling procedure using a handheld glucometer (Accu-Chek, Roche, Basel, Switzerland). Blood samples in the EDTA tubes were centrifuged at 1400× *g* at 4 °C for 15 min. Plasma supernatant was extracted and stored in aliquots (120 μL) in microcentrifuge tubes (Axygen, Corning Life Sciences, New York, United States) at –80 °C for analysis of concentration of FGF21, oxLDL and adiponectin.

### 2.3. Arterial Stiffness Parameters

Arterial stiffness was measured indirectly using PWV between the carotid and femoral arteries (SphygmoCor XCEL, AtCor Medical Pvt, Ltd., NSW, Sydney, Australia). Blood pressure cuffs were wrapped around the right upper thigh (femoral artery) with the participants wearing comfortable casual attire. The participants rested quietly in a supine position for 15 min before measurements of arterial pulse pressure waveforms started. PWV was measured simultaneously with pressure transducers, by acquiring a carotid pulse by applanation tonometry and a femoral pulse by volumetric displacement, within an inflated cuff around the upper thigh. The pulse waves were captured electronically on a computer using the SphygmoCor system and accepted by the system after consistent high-quality waveforms were measured. An average of approximately three to five measurements were taken.

### 2.4. Bioassays

Plasma FGF21 concentration was measured with undiluted samples using a commercially available enzyme-linked immunosorbent assay (ELISA) according to the instructions of the manufacturer (DF2100; R&D Systems, Inc., Minneapolis, MN, USA). Plasma adiponectin concentration was measured with 500-fold dilution of the plasma samples, using the Adiponectin Human ProcartaPlex Simplex kit (eBioscience, Affymetrix, Vienna, Austria) according to the manufacturer’s instructions. The assay samples were read on the Bio-Plex 200 System (Bio-Rad Laboratories, Inc, Hercules, CA, USA) and the concentrations for each well were analysed with the Bio-Plex Manager Software (Version 6.1, Bio-Rad Laboratories, Inc, Hercules, CA, USA). Human plasma oxLDL concentrations were measured with 6561-fold dilution of plasma samples, using a commercially available ELISA kit according to the manufacturer’s instructions (Mercodia oxLDL ELISA, Uppsala, Sweden). Intra- and inter-assay variation for our assays were <5% for FGF21 and adiponectin concentrations and <6% for oxLDL concentration.

### 2.5. Statistics

All statistical analyses were performed using IBM Statistical Package for Social Sciences (SPSS), version 23 (IBM Corp., Armonk, NY, USA). A sample size of 80 participants (20 per group) was needed for the trial to have 80% power to detect a two-sided hypothesis test at an α level of 0.05 (effect size of 0.64) (G*Power, version 3.1, Dusseldorf, Germany). Numerical variables are presented as means and standard deviations (SD). The participant characteristics were analysed using two-way analysis of variance (ANOVA), with age, exercise and age × exercise as fixed effects, to assess potential differences between age and exercise groups at baseline. PWV and blood-related parameters were also analysed using two-way ANOVA (Age × Exercise). The normality and heterogeneity of variance were tested using the Shapiro–Wilk test and Levene’s test respectively. To adjust for potential confounding factors, sex was analysed as a categorical control variable, and BMI and WC were treated as continuous predictor variables in the two-way ANOVA analyses. Plasma FGF21, plasma adiponectin and PWV data did not meet the criteria for normal distribution. Logarithmic transformation was performed before analysis for PWV data and plasma FGF21 concentrations. The data for plasma adiponectin concentration was transformed using the cube-root function. Pearson’s correlation was used to evaluate associations between two variables. A partial correlation model was used to adjust for potential confounding variables, such as sex or weekly exercise duration, in the correlation analysis between two variables. Some of the sedentary participants did not engage in weekly exercise, resulting in clustering of data at the low end for weekly exercise duration. Weekly exercise duration was thus stratified into four groups based on 0 min (*n* = 26), <180 min (*n* = 19), 180–360 min (*n* = 19) and >360 min (*n* = 16), to ensure sufficient participants within each group and an even distribution of participants across the groups. One-way ANOVA was used to analyse the relationships between weekly exercise duration groups with PWV and oxLDL concentration. To adjust for confounding factors, age was treated as a continuous variable and sex was analysed as a categorical control variable in the one-way ANOVA model. All data in the tables and figures are presented using the original untransformed data. Statistical significance was accepted if *p* < 0.05.

## 3. Results

### 3.1. Participant Demographics, Anthropometry, Blood Pressure, Lipids and Glucose

The demographic, physical and blood pressure profiles of the eighty participants are presented in Table 1. Age was equally distributed between active and sedentary participants in both younger and older groups. Sex was equally matched between the YA and YS group and there was one more male participant in the OA than in the OS group. There were no significant differences in body mass and height among different age and exercise groups. However, BMI was significantly lower in the younger than the older group (*p =* 0.003), independent of exercise status. Compared with younger participants, older participants also had higher WC (*p* < 0.001), independent of exercise habits (Table 1). Compared with the younger participants, older participants had higher systolic (10%) and diastolic (12%) BP (all *p* < 0.001), regardless of exercise habits. Diastolic BP was 6% lower in active than sedentary groups (*p* < 0.006) (Table 1). TC, LDL-C and TG concentrations were higher in older than younger adults (all *p* < 0.001), while HDL-C was higher (*p* = 0.012), and TG was lower in active than in sedentary individuals (*p* = 0.003) (Table 1). Fasted blood glucose concentrations were higher in older than younger adults (*p* = 0.043) (Table 1).

### 3.2. Associations Between Habitual Exercise and Ageing with PWV and oxLDL

**Figure 1:**
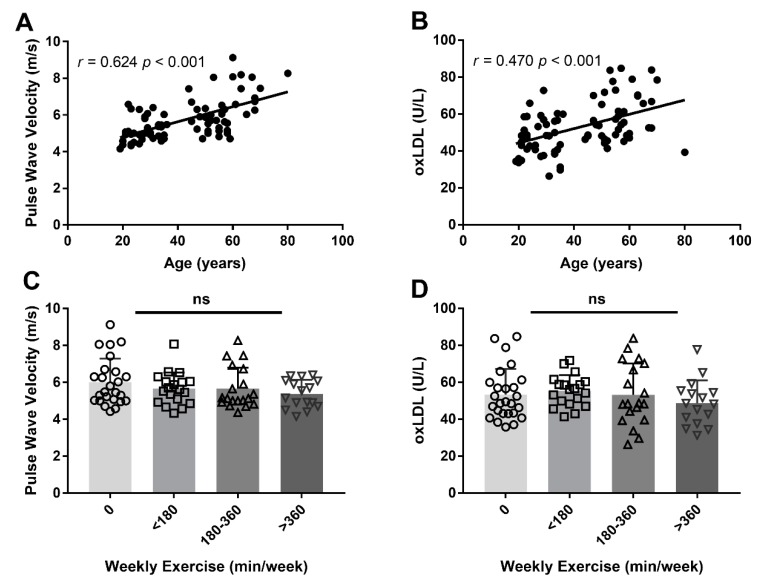
Pearson’s correlation between age with pulse wave velocity (PWV) (**A**), as a surrogate marker for arterial stiffness, and oxidised low-density lipoprotein (oxLDL) concentrations (**B**). One-way analysis of variance was conducted for PWV (**C**) and oxLDL concentrations (**D**) between weekly exercise duration, stratified across four groups. Each symbol represents an individual that participated in 0 min (circle), <180 min (square), 180–360 min (upward triangle) and >360 min (downward triangle) of weekly exercise for the past five years. ns = not significant.

Age was positively correlated with PWV (*r* = 0.624, *p* < 0.001, Figure 1A) and with plasma oxLDL concentration (*r* = 0.470, *p* < 0.001, Figure 1B), even after adjusting for weekly exercise duration (*r* = 0.637, *p* < 0.001) and sex (*r* = 0.461, *p* < 0.001) in a partial correlation analysis. PWV (*p* = 0.340) and oxLDL concentrations (*p* = 0.536) did not differ across the weekly exercise duration groups (Figure 1C,D), even after adjusting for sex (*p* = 0.185) and age (*p* = 0.373).

### 3.3. Associations of Exercise and Ageing with FGF21, Adiponectin, Oxidative Stress and PWV

**Figure 2:**
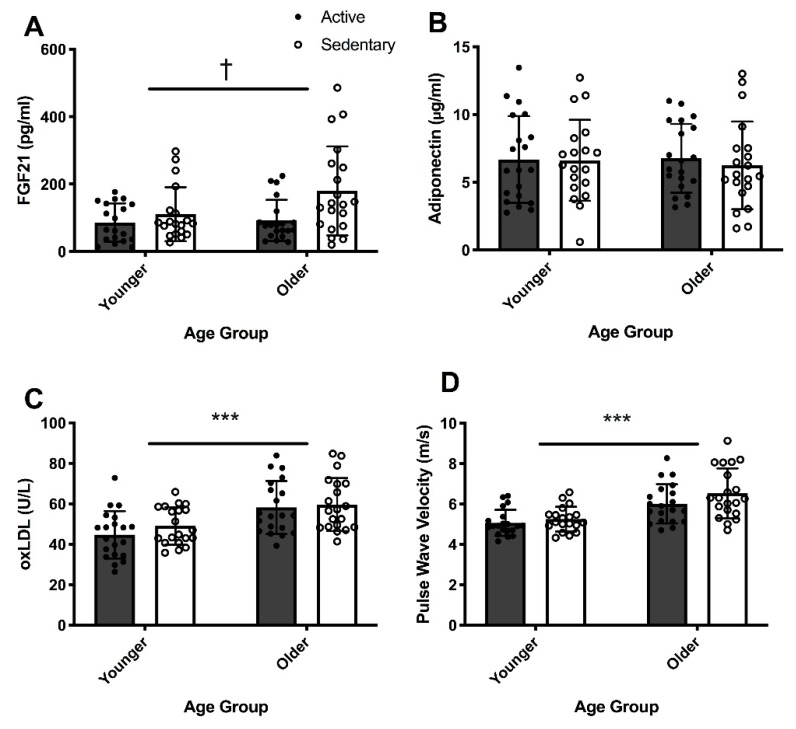
Two-way analysis of variance (age × exercise) of fibroblast growth factor 21 (FGF21) (**A**), adiponectin (**B**), oxidized low-density lipoprotein (oxLDL) (**C**) concentrations and pulse wave velocity (PWV) (**D**) in younger active (YA) (*n* = 20, M/F = 10/10) and sedentary (YS) (*n* = 20, M/F = 10/10), and in older active (OA) (*n* = 20, M/F = 11/9) and sedentary (OS) (*n* = 20, M/F = 12/8) individuals. Two-way ANOVA for FGF21 concentration was conducted only for YA (*n* = 19, M/F = 10/9), YS (*n* = 19, M/F = 9/10), OA (*n* = 20, M/F = 11/9) and OS (*n* = 20, M/F = 12/8) groups *** = *p* < 0.001 for age. † = *p* < 0.05 for exercise status. Black circles represent active individuals and white circles represent sedentary individuals within younger and older groups.

Plasma samples from two participants were insufficient for determination of FGF21 concentration and were excluded from the analysis. The FGF21 concentrations reported are from 78 participants: YS (*n* = 19), YA (*n* = 19), OS (*n* = 20), OA (*n* = 20). Circulating FGF21 concentration was not different between older and younger groups, regardless of exercise habits (*p* = 0.097), even after adjusting for sex, BMI and WC (*p* = 0.397) (Figure 2A). However, fasted circulating concentration of FGF21 was 9% lower in active participants than their sedentary counterparts regardless of age (*p* = 0.011), even after adjusting for sex (*p* = 0.012) (Figure 2A). The interaction effect of age and exercise was not significant for plasma FGF21 concentration (*p* = 0.475) (Figure 2A).

Circulating adiponectin concentration was not significantly different between active and sedentary groups (*p =* 0.821, Figure 2B) or between younger and older groups (*p* = 0.642, Figure 2B), even after adjusting for sex, BMI and WC (*p* = 0.399). No significant interaction effect was observed for plasma adiponectin concentration (*p* = 0.418) (Figure 2B).

Compared with their younger counterparts, older adults had 25.6% higher basal plasma oxLDL concentrations (*p* < 0.001), even after adjusting for sex, BMI and WC (*p* = 0.001) (Figure 2C). Habitual exercise was not associated with oxLDL concentrations (*p* = 0.275), even after adjusting for sex (*p* = 0.259). There was no significant interaction effect of age and exercise for oxLDL concentration (*p* = 0.583) (Figure 2C).

Compared with the younger participants, the PWV in older participants was 0.2-fold higher (*p* < 0.001, Figure 2D). Adjusting for sex, BMI and WC did not influence these differences observed in PWV with age. However, PWV was not statistically different between active and sedentary groups (*p* = 0.09), even after adjusting for sex (*p* = 0.065, Figure 2D). No significant interaction between age and exercise was observed for PWV.

### 3.4. Correlations Between FGF21, Adiponectin, oxLDL and PWV

When analysed as an entire cohort, the correlation between plasma FGF21 concentration and PWV was not statistically significant (*r* = 0.220, *p =* 0.054, *n* = 78, Table 2) and this relationship did not change in subgroup analyses conducted by age and exercise habits. Adjusting for sex in a partial correlation analysis between FGF21 concentration and PWV was statistically significant (*r* = 0.232, *p* = 0.044). In contrast, higher plasma adiponectin concentration was negatively correlated with PWV (*r* = –0.384, *p* < 0.001, *n* = 80, Table 2). Plasma oxLDL concentration was positively correlated with PWV (*r* = 0.367, *p* = 0.001, *n* = 80, Table 2). When analysed as an entire group, there were no significant correlations between plasma FGF21, adiponectin or oxLDL concentrations (all *p* > 0.05, *n* = 80, data not shown) and adjusting for sex in a partial correlation model did not affect the results presented.

For subgroup analysis, plasma adiponectin concentration was negatively correlated with PWV within the younger group (*r* = –0.359, *p* = 0.025, *n* = 40, Table 2). However, there was a stronger correlation between adiponectin and PWV in the older group (*r* = –0.509, *p* = 0.001, *n* = 40, Table 2), when examining all activity groups together. In subgroup analysis, plasma oxLDL concentration was also positively correlated with plasma FGF21 concentration in the older (*r* = 0.345, *p* = 0.029, *n* = 40), but not in the younger (*r* = –0.132, *p* = 0.431, *n* = 38) group, even after adjusting for sex in a partial correlation model, independent of activity status.

In subgroup analysis, plasma adiponectin concentration was negatively correlated with PWV in the sedentary participants (*r* = –0.511, *p* = 0.001, *n* = 40), but not in active participants (*r* = –0.198, *p* = 0.220, *n* = 40), when examining all ages together (Table 2). In contrast, in active individuals, a positive correlation between plasma oxLDL concentration and PWV was found (*r* = 0.452, *p* = 0.003, *n* = 40) (Table 2). Adjusting for sex in a partial correlation analysis did not affect these subgroup correlations in the sedentary and active groups.

## 4. Discussion

The present study investigated the associations between ageing and exercise habits with oxidative stress and a surrogate measurement of arterial stiffness, PWV, in healthy individuals. To our knowledge, this study is the first to explore the relationship between the FGF21- and adiponectin-associated pathway with oxidative stress and PWV. Compared with younger individuals, older individuals had higher circulating plasma oxLDL concentrations and higher PWV. These results are consistent with previous findings that ageing independently increases oxidative stress by 1.2–3-fold and PWV by ~2-fold [2,31,32]. In our study, oxLDL concentration was positively correlated to PWV. These results extend the current understanding that an age-related increase in oxidative stress is associated with arterial stiffening even in healthy individuals. Consistent with our results, oxidative stress was independently and positively associated with PWV in middle-aged and older adults, regardless of health status in previous studies [33,34]. Oxidative stress contributes to arterial stiffness by decreasing endothelial nitric oxide synthesis and bioavailability, which impairs arterial compliance and increases arterial stiffness [35,36]. Compared to younger adults, healthy elderly individuals had increased oxLDL concentration, which was negatively related to forearm blood flow when subjected to reactive hyperaemia, suggesting that age-associated increase in oxidative stress influenced vascular function and arterial stiffness [32].

Plasma FGF21 concentrations were not different between the age groups in our study, implying that the age-associated increase in oxidative stress was unlikely mediated by FGF21. In contrast, earlier findings suggested that FGF21 concentration increased with age in healthy individuals (aged 5–80 years) [25], and was involved in the regulation of oxidative stress [15,16,17]. Moreover, increased intracellular stress signalling due to oxLDL administration resulted in increased FGF21 expression in animal cardiac cells and hepatocytes [16,17]. Administration of FGF21 also protected against oxidative stress in human endothelial cells, supporting the antioxidative role of FGF21 in improving PWV [15]. The positive associations of FGF21 with arterial stiffness in male and female patients on haemodialysis [12] and patients with type 2 diabetes [13], and in obese women [37], could also suggest the compensatory antioxidative effects of FGF21 on increasing arterial stiffness. Collectively, these earlier results support the role of FGF21 in mediating an age-associated increase in arterial stiffness by decreasing oxidative stress. The different findings in our study could be due to the participant inclusion criteria, which was limited to a clinically healthy (free from metabolic and cardiovascular diseases) and non-obese population. The antioxidant and cardio-protective effects of FGF21 could be more obvious in diseased populations, who have higher oxidative stress and higher fasted circulating FGF21 concentrations [11,38]. In addition, most of the mechanistic studies involving FGF21 and oxidative stress were done on animal or in vitro models, which are difficult to translate to the healthy participants in our study. Contrary to the associated increase in antioxidant activity with FGF21 in attenuating arterial stiffness [15,16,17], the present study showed no age-associated differences in FGF21 concentration in healthy young and older adults. Our results appear to suggest that FGF21 is unlikely involved in age-associated increases in oxidative stress and arterial stiffness. Our findings differed from earlier studies reporting an age-associated increase in FGF21 concentration [25,39]. This difference in results could be due to differences in methods of data analysis and profiles of subjects. For example, one study included participants from five to 80 years old, and analysed age as a categorical variable in five groups [25], while participants in another study were 90–100 years old, with heterogenous health status, such as surgery for hip dysplasia [39]. The profile of these subjects differed from our study, who were all healthy and with age ranging from 18–36 in the younger group, and from 45–80 in the older group. We compared FGF21 concentrations between younger and older groups, rather than a multiple linear regression [25] or correlation analyses [39]. Our sample size is also relatively small compared with earlier studies that achieved a positive association between FGF21 concentration and age [25,39]. The borderline significant positive correlation between FGF21 concentration and PWV suggests that increased circulating FGF21 concentration or basal FGF21 resistance may be related to higher arterial stiffness, perhaps through anti-inflammatory mechanisms rather than antioxidative pathways. Instead of the direct FGF21-associated effects on antioxidant pathways with arterial stiffness, FGF21 may also indirectly affect arterial stiffness through oxLDL-induced pyroptosis and related cellular dysfunction through specific receptors [40].

In this study, the concentration of the FGF21 downstream effector, adiponectin [19,41], was negatively correlated with PWV, suggesting that adiponectin may be linked to lower arterial stiffness even in healthy individuals, independent of FGF21. Adiponectin could improve arterial stiffness through an increase in proteins involved in antioxidant pathways [24]. In mice, adiponectin decreased systemic oxidative stress and normalised endothelial cell function through increased nitric oxide production, which could, in turn, decrease arterial stiffness [42,43]. In humans, an inverse relationship was also reported between plasma adiponectin concentration and PWV, supporting the potential role of adiponectin in improving arterial stiffness [44]. In the sub-group correlation analyses, the negative correlation between plasma adiponectin concentration and PWV was more apparent in the older than the younger participants, and in the sedentary than the active participants. Plasma FGF21 concentration was also positively correlated with oxLDL concentration only in the older but not the younger group. Our preliminary findings imply that FGF21 and adiponectin may independently act as compensatory responses to mitigate oxidative stress and arterial stiffness, respectively, especially in older and sedentary adults. These findings also suggest that different mechanisms may drive arterial stiffness in young and older adults. For example, in adults < 50 years old, age-associated arterial stiffness was due to an increase in the magnitude of wave reflection in peripheral arteries, whereas in individuals >50 years old, age-associated arterial stiffness was due to increased wave velocity resulting from a less compliant central aorta [2,31]. Taken together, our results suggest that adiponectin may regulate age-associated arterial stiffening, independent of FGF21, especially in healthy older adults.

The plasma concentration of oxLDL and PWV in the present study were not different between habitually active and sedentary groups, suggesting that habitual exercise may not affect oxidative stress and arterial stiffness. However, others have reported a negative association between habitual exercise participation and arterial stiffness in healthy young adults [45,46]. Short-, medium- and long-term exercise have been found to reduce arterial stiffness in younger populations. For example, six days of endurance training at 65% of peak oxygen consumption in young healthy males (~25 years old) reported an 8% improvement in PWV [46] and eight weeks of aerobic exercise at 60–80% heart rate reserve also improved PWV in both healthy males and females (~41 years old) [47]. A 24-year longitudinal study in healthy males and females (starting from 13 years old) demonstrated that long-term vigorous habitual physical activity was associated with improved arterial stiffness [45]. A six-week exercise and dietary intervention in children who were obese also reduced oxLDL concentration [48]. These results were also found in adult men and women, aged 18–65 years, where 4 weeks of moderate intensity exercise decreased oxLDL concentration by 14% [49]. These earlier findings suggest that habitual exercise improves arterial stiffness regardless of age and that exercise-induced improvement in arterial stiffness could be mediated by a reduction in oxidative stress. The discrepancies in earlier findings which reported improvements in arterial stiffness with exercise were done mostly on only younger [45,46] or middle-aged [47] adults, which differed from the 45–80 (older group) age group in our study. The effects of exercise on attenuating arterial stiffness may be age-dependent, as longer physical activity duration decreased PWV by 7% only in older adults aged > 60 years [50]. The favourable effects of exercise on arterial stiffness may be more apparent for higher intensity exercises [46,47], unlike in our study, which included moderate to high intensity exercises. The history of exercise duration and intensity for active participants was documented using a self-reported approach, which differed from other studies with controlled exercise interventions, objectively quantified exercise intensities [46,47] and accelerometery-measured physical activity [50]. Our cross-sectional study design could have contributed to the disparity in results with earlier longitudinal studies [45] or intervention studies, which administered chronic exercise bouts [48,49]. Our study compared PWV between habitually active and sedentary individuals, while earlier exercise intervention studies recruited participants of various activity profiles including <2 h/week of low to moderate intensity exercise [46] or VO_2max_ ~24 mL/min/kg [47]. These differences in study design, study population and exercise intensity could have contributed to the disparity between our results and results that were reported previously on habitual exercise with oxidative stress and arterial stiffness.

A key limitation of the present study is the cross-sectional design, which could demonstrate associations but not prove causality between higher oxLDL and FGF21 concentrations or lower adiponectin concentrations, and the state of arterial stiffness. Given that plasma oxLDL concentration was measured as a surrogate marker of oxidative stress, the exact mechanisms driving an association between oxidative stress and PWV cannot be determined from this study. Future studies should investigate the effects of FGF21 on nitric oxide and endothelial function as potential pathways in mediating arterial stiffness. We did not account for cysteine or the pro-inflammatory cytokines and chemokines of the participants in the present study, which could have affected the FGF21 and oxidative stress concentrations measured. These present findings are based on our inclusion criteria of healthy, non-smokers, non-habitual alcohol drinkers and females who were not on oral contraceptives or hormone replacement therapy. These inclusion criteria were designed to isolate the effects of habitual exercise and ageing on the parameters studied, but the results may not be generalized or representative of other biomarkers and populations. Future studies could possibly recruit individuals who are more representative of the general population. This study provided useful early data on the potential links between oxidative stress, FGF21 and adiponectin with age-related arterial stiffness. However, as the current sample size is relatively small, future studies involving a larger elderly cohort are needed to shed more light on the mechanistic pathways regulating the interactions on oxidative stress, FGF21, adiponectin and arterial stiffness with age. Future studies could also administer exercise interventions and quantify exercise intensity using more objective methods in order to better investigate the effects of exercise on FGF21, oxidative stress and arterial stiffness. Understanding the underlying mechanisms of oxidative stress-associated arterial stiffness can potentially contribute to the development of therapeutic targets to improve arterial compliance and reduce cardiovascular risk.

## 5. Conclusions

In summary, this study demonstrated that ageing is associated with an increase in oxidative stress and arterial stiffness, but this is unlikely mediated through FGF21 and adiponectin. Habitual exercise unlikely attenuates oxidative stress or arterial stiffness in healthy individuals.

## Figures and Tables

**Table 1:** Participant demographics, exercise history, anthropometry and blood pressure.

	Young Sedentary (YS)	Young Active (YA)	Older Sedentary (OS)	Older ActiveV (OA)	Age	Exercise	Age × Exercise
*n*	20	20	20	20			
Sex *n* (M/F)	10/10	10/10	11/9	12/8			
Exercise intensity	Light	Mod-High	Light	Mod-High			
Exercise (min/week)	18 (27)	369 (177)	16 (28)	350 (214)	0.747	**<0.001**	0.790
Age (years)	28 (5)	28 (5)	56 (7)	57 (9)	**<0.001**	0.668	0.668
Body mass (kg)	59.3 (7.2)	60.4 (13.8)	64.3 (13.5)	62.9 (11.8)	0.150	0.937	0.619
Height (cm)	169.6 (8.2)	166.8 (10.1)	163.9 (8.7)	167.5 (8.6)	0.208	0.852	0.116
BMI (kg/m^2^)	20.6 (2.2)	21.5 (2.2)	23.8 (4.0)	22.2 (2.4)	**0.003**	0.563	0.053
WC (cm)	72.9 (5.3)	72.8 (7.0)	82.1 (9.2)	77.8 (8.5)	**<0.001**	0.207	0.216
Systolic BP (mmHg)	109 (8)	110 (5)	121 (11)	119 (12)	**<0.001**	0.891	0.577
Diastolic BP (mmHg)	67 (7)	64 (5)	76 (8)	70 (8)	**<0.001**	**0.006**	0.247
PP (mmHg)	38 (9)	39 (9)	38 (8)	42 (12)	0.500	0.229	0.469
TC (mmol/L)	5.0 (0.6)	4.8 (1.1)	5.6 (0.7)	5.9 (0.9)	**<0.001**	0.760	0.202
HDL-C (mmol/L)	1.6 (0.3)	1.9 (0.5)	1.6 (0.5)	1.9 (0.4)	0.988	**0.012**	0.707
LDL-C (mmol/L)	3.0 (0.6)	2.6 (0.7)	3.4 (0.6)	3.5 (0.8)	**<0.001**	0.452	0.082
TG (mmol/L)	0.8 (0.2)	0.7 (0.2)	1.2 (0.4)	1.0 (0.4)	**<0.001**	**0.003**	0.944
Fasted glucose (mmol/L)	4.8 (0.3)	4.9 (0.4)	5.0 (0.3)	5.0 (0.4)	**0.043**	0.583	0.814

Body mass index (BMI), waist circumference (WC), blood pressure (BP), pulse pressure (PP), total cholesterol (TC), High density lipoprotein cholesterol (HDL-C), low density lipoprotein cholesterol (LDL-C), triglyceride (TG). Mod = moderate. Values are mean (SD). *p* values are indicated in the last three columns and statistically significant values are indicated with a bold type interface.

**Table 2:** Pearson’s correlations (*r*) between FGF21, adiponectin, oxLDL and pulse wave velocity (PWV).

	*n*	M/F	Biomarker	PWV
Total	78	42/36	**FGF21**	0.220 (0.054)
	80	43/37	**Adiponectin**	**–0.384 (<0.001)**
	80	43/37	**oxLDL**	**0.367 (0.001)**
Younger	38	19/19	**FGF21**	–0.081 (0.634)
	40	20/20	**Adiponectin**	**–0.359 (0.025)**
	40	20/20	**oxLDL**	0.157 (0.339)
Older	40	23/17	**FGF21**	0.262 (0.103)
	40	23/17	**Adiponectin**	**–0.509 (0.001)**
	40	23/17	**oxLDL**	0.174 (0.283)
Active	39	21/18	**FGF21**	0.083 (0.614)
	40	21/19	**Adiponectin**	–0.198 (0.220)
	40	21/19	**oxLDL**	**0.452 (0.003)**
Sedentary	39	21/18	**FGF21**	0.272 (0.099)
	40	22/18	**Adiponectin**	**–0.511 (0.001)**
	40	22/18	**oxLDL**	0.259 (0.111)

Values are unadjusted Pearson’s *r* on top and *p* values in brackets below. Values in bold type interface represent statistically significant correlations.
